# Process and outcome of child psychotherapies offered in Kenya: a mixed methods study protocol on improving child mental health

**DOI:** 10.1186/s12888-020-02611-2

**Published:** 2020-05-27

**Authors:** Grace Nduku Wambua, Manasi Kumar, Fredrik Falkenström, Pim Cuijpers

**Affiliations:** 1 Department of Clinical, Neuro and Developmental Psychology, Amsterdam Public Health Research Institute, Vrije Universiteit Amsterdam, Amsterdam, The Netherlands; 2 Department of Psychiatry, University of Nairobi, Nairobi, Kenya; 3 Department of Behavioral Sciences and Learning, Linköping University, Linköping, Sweden

**Keywords:** Child and adolescent mental health, Psychotherapy, Outcomes, Process

## Abstract

**Background:**

Child and adolescent mental health problems account for a significant proportion of the local and global burden of disease and is recognized as a growing public health concern in need of adequate services. Studies carried out in Kenya suggest a need for a robust service for the treatment, prevention, and promotion of child and adolescent mental health. Despite a few existing services to provide treatment and management of mental health disorders, we need more knowledge about their effectiveness in the management of these disorders. This paper describes a study protocol that aims to evaluate the process and outcomes of psychotherapies offered to children and adolescents seeking mental health services at the Kenyatta National Hospital in Kenya.

**Methods:**

This study will use a prospective cohort approach that will follow adolescent patients (12–17 years of age) receiving mental health services in the youth clinics at the Kenyatta National Hospital for a period of 12 months. During this time a mixed methods research will be carried out, focusing on treatment outcomes, therapeutic relationship, understanding of psychotherapy, and other mental health interventions offered to the young patients. In this proposed study, we define outcome as the alleviation of symptoms, which will be assessed quantitatively using longitudinal patient data collected session-wise. Process refers to the mechanisms identified to promote change in the adolescent. For example, individual participant or clinician characteristics, therapeutic alliance will be assessed both quantitatively and qualitatively. In each session, assessments will be used to reduce problems due to attrition and to enable calculation of longitudinal change trajectories using growth curve modeling. For this study, these will be referred to as session-wise assessments. Qualitative work will include interviews with adolescent patients, their caregivers as well as feedback from the mental health care providers on existing services and their barriers to providing care.

**Conclusion:**

This study aims to understand the mechanisms through which change takes place beyond the context of psychotherapy. What are the moderators and through which mechanisms do they operate to improve mental health outcomes in young people?

## Background

Children and adolescents make up more than a third of the global population, and just over 50 % of the people in low- and middle-income countries (LMICs) [1]. Child and adolescent mental health problems account for a significant proportion of the global burden of disease [2] and is a growing public health concern. A meta-analysis by Polanczyk et al. [3] found a global prevalence of 13.4% of children and adolescents affected by any mental disorder: depression, anxiety, and post-traumatic stress disorders as leading mental health disorders [4, 5]. In sub-Saharan Africa, an estimated 10–20% of children and adolescents need some form of mental health services [6, 7]. Despite the emergence of policies and guidelines there is still a need to understand the contextual factors that play a role in the development and implementation of Child and Adolescent Mental Health (CAMH) policies, services, and programs [8, 9]. Children and adolescents in LMIC contexts are greatly affected by the gap that exists between the need for and the access to mental health services, with the treatment gap estimated to be as high as 90% [1].

Prevalence studies from various settings have documented significant psychiatric morbidity in children and adolescents in Kenya with affective and conduct disorders being the most prevalent and most affected patients being found in urban areas [10–16]. Findings from these studies highlight the need to establish child mental health services for the child and adolescent population in the country. Unfortunately, as is the case in many LMICs, the treatment gap in CAMH in Kenya is hampered by limited services, as well as limited human and economic resources [17, 18]. The treatment and management of CAMH problems differ from that given to adults, given that their developmental needs are different. Thus, young patients require specialized attention. It is established that the human resources available possess limited skills and knowledge for providing quality mental health services for this population; this is primarily due to lack of access to adequate CAMH focused training [16, 19, 20].

The existence of health policies and plans ensure that systems of care are not fragmented, ineffective, expensive, or inaccessible. Mental health policy in Kenya is lagging. The Kenya Mental Health Act 1989 is now outdated, resulting in the lack of a comprehensive national mental health law [21]. The Kenya Mental Health Bill of 2014 was introduced and is awaiting review in Parliament. The 2010 Kenyan Constitution includes a Bill of Rights that guarantees everyone—including those with children and adolescents with CAMH disorders—certain rights, including the right “to the highest attainable standard of health, which includes the right to health care services and the right to emergency medical treatment” [22]. Other relevant national policies exist and include, the Kenya Health Policy 2014–2030, the Kenya Mental Health Policy 2015–2030 (launched in 2015) [23], and the Health Sector Strategic and Investment Plan all of which include strategies to address mental health care [20]. Policies and frameworks for children, such as the National Children Policy Kenya 2010 [24] and National Plan of Action for Children in Kenya 2015–2022 [25] cater to the needs of this population. Still, they do not include mental health as an essential priority for the child. Despite the identification of this group as a vulnerable population, CAMH problems are not prioritized in the country.

Mental health research in Kenya has come a long way. Still, when it comes to children and adolescents, it is undeniable that there is a need to develop a more robust base for CAMH epidemiological and service research. Significant gaps still exist when it comes to an understanding of the effectiveness of treatments used for the management of child mental disorders, as well as what factors influence the processes involved.

Empirical evidence shows that childhood disorders are amenable to treatment [26, 27], with research showing psychological interventions to be useful for the amelioration of symptoms and impairment associated with child and adolescent mental health problems. Effectiveness of an intervention describes as the probability that it will produce beneficial effects for typical clients, treated by the average practitioner, under ordinary clinical practice conditions [28, 29]. It is assessed by improved outcomes, which are the results of the therapy. Several meta-analyses point to beneficial, problem-specific, and long-lasting effects of mental health interventions for a variety of child conditions [26]. Another important aspect of treatment is the therapeutic process, described as what takes place between, and within, the patient and therapist during their interaction [30, 31]. Process in psychotherapy research refers to how psychotherapy produces its effects and is often linked to outcome [32, 33]. The process is crucial as it helps researchers understand both the internal and external factors that mediate between the social environment and the child’s emotional experience and behavior, influencing the outcomes of the intervention. An essential aspect of the therapy process is the therapeutic alliance, which refers to the relationship between patient and therapist [34]. It is a collaborative relationship that requires confidence, trust, and positive regard between the two. The relationship promotes a sharing of beliefs regarding the goals of treatment, methods used as well as the patient’s faith in the therapist’s ability to help them and the therapist’s confidence in the patient’s resources. This alliance may influence the outcome as it enables the patient to accept, follow, and believe in the treatment.

Evaluations of interventions vary, with some focusing on efficacy, effectiveness, client outcomes, or cost [35]. Reviews show positive results for psychosocial interventions with children and adolescents [36–38]. Little is known about methods to evaluate treatment outcomes and processes from both client and therapist perspectives in LMIC contexts, including Kenya. Self-rating scales used for treatment evaluation in the West found to be valid and reliable are now used in LMIC settings. A study with adults in the Kenyan context also made use of various self-reporting questionnaires to assess treatment processes and outcomes [39]. Findings from this study showed that the participants reported improvement in their mental health condition, and distress reduced as psychotherapy progressed. A gap still exists when it comes to assessing treatment outcomes and processes in children and adolescents.

This study, therefore, aims to fill that gap by evaluating the process and outcomes of psychotherapies offered to adolescents seeking mental health services at the Kenyatta National Hospital, – the largest referral hospital in Kenya and one of the few hospitals with dedicated child and adolescent mental health clinics. This study will address the following questions:

1. Is there any improvement, as evidenced by improved outcome scores, in adolescents receiving psychotherapy in routine clinical service offered to them in a public hospital?
2. Is there a relationship between the therapeutic alliance and clinical outcomes in adolescents receiving treatment?
3. What is the client’s (adolescent and caregiver’s) views on mental health treatment and services in alleviating their difficulties?
4. What are the client’s and therapist’s perceptions of the therapeutic process?

## Methods

### Study design

This study will make use of a naturalistic prospective cohort design to track the process and outcomes associated with psychotherapies. It will follow a cohort of patients who receive psychotherapy for 1 year. Patients will undergo treatment as usual, with no interference from the researcher except for the session-wise assessments. Treatment, as usual in this context, varies from clinician to clinician. The process starts with a thorough clinical intake assessment from either a student or trainee psychologist (counselling/clinical) or a psychiatric nurse with the supervision of the on-call consultant psychiatrist to decide clinical diagnosis and treatment plan, and this may take one-two sessions. Face-to-face follow-up sessions vary, with five being the minimum number of sessions that require attendance. The clinicians are trained in evidence-based treatments, which they often adapt to the client’s needs. Clinicians may take either a supportive or skill-based approach depending on the patient’s presenting complaints. Therapy type and focus, as well as the number and frequency of sessions, vary from patient to patient.

Data will be collected from the participants during every session to reduce problems due to attrition and to enable calculation of longitudinal change trajectories using growth curve modeling. Process and outcomes tracked from a bi-directional point of view with adolescents, caregivers, and clinicians giving feedback on a session-wise basis.

### Study setting and study population

This study will take place at the Kenyatta National Hospital, Kenya’s National training, and referral hospital. The outpatient adolescent (youth) clinic forms part of the Mental Health Department and will be the clinic of focus for this study. This clinic offers counseling and psychotherapy services to patients referred for any psychiatric or psychological problems from within and outside the hospital.

The clinic sees an average of 533 patients monthly, with affective and conduct disorders being the most prevalent. The clinic caters to patients between the ages of 12–24 years referred from within and around Nairobi County. Being a referral hospital, from time to time, the clinic may receive referrals of adolescents from other counties. Socio-demographics of the patients and their caregivers are also diverse. These are some of the information we will find out in our demographic questionnaire. The services offered at the clinic vary from patient to patient and are dependent on their presenting issues. The services may differ from grief counseling, motivational interviewing, engagement interviewing, brief solution therapy, cognitive behavioral therapy, trauma-focused cognitive behavioral therapy, and interpersonal therapy among others. The clinic has three consultant psychiatrists attached to it. During a patient’s intake, the clinicians carry out structured diagnostic interviews, and together with the supervision of the psychiatrists, they will give a current Diagnostic and Statistical Manual of Mental Disorders version five (DSM 5) diagnosis where applicable.

The study aims for a total population study of the clinic (within the limits of informed consent and assent), and the researcher seeks for minimum participation of 80% to ensure that generalization to the studied clinic will not be biased. With that in mind, the study will use a cross-sectional design Cochrane formula [40] to determine the minimum required sample size population, using a sample frame of 533 persons (confidence level of 95%, a precision of 5%). The minimum sample size for the proposed study is 224, but the researchers will recruit 260 to help counter the possibility of panel attrition.

Adolescents between the ages of 12–17 years seeking services at these clinics, with moderate to severe impairment and with a current DSM5 diagnosis, will be included in the study. We will invite both caregivers and clinicians of these adolescents to participate in the study.

### Recruitment and consenting procedures

We will approach caregivers with adolescents attending the clinic after they have seen their assigned clinician (psychotherapist/counselor/psychiatrist) for the first time. The clinicians providing services to recruited adolescents will also be required to provide written consent to participate in the study. The researcher or research assistant will fully disclose the purposes and components of the study. They will discuss with them any potential risks of participating in the study. Those who require clarification on the nature and purpose of the study will be on a need-by-need basis. Refusal to participate in the study will in no way influence the services they receive at the clinics. There will be no rewards for participation.

### Data collection instruments

This study will make use of multiple assessments. As the investigation is longitudinal, many of the measures will be repeated from session to session.

1. *Demographic questionnaire****-*** The author has designed a survey that will capture the relevant demographic variables. Information such as age, gender, family type (two-parent or single-parent home, living with a guardian), parental level of education, reasons for referral, past and present psychiatric history (have they had prior visits to a counselor, psychologist or psychiatrist), medical history, family and social history (any family member with past or present psychiatric history, suicidal deaths, substance use/abuse). Information on DSM5 diagnosis and proposed therapy will also be retrieved. This questionnaire will also have a section for the clinicians to give information about age, gender, level of education, educational focus (counseling/clinical) and additional training, number of years practicing, their clinical diagnosis of the adolescent. The adolescent, caregiver, and clinician will fill this questionnaire.
2. *Paediatrics Symptom Checklist (PSC)* [41] ***–*** The PSC is a screening measure used to asses child and adolescent psychosocial well-being. It is used to identify individuals who may need further evaluation, or as an indicator of psychosocial well-being before and following intervention/treatment. Both parents (PSC - 6-16 yrs), and the adolescents themselves will fill the youth version (Paediatrics Symptom Checklist - Youth, PSC-Y) (11 + years). It has 35 items assessing internalizing, externalizing, and attention problems. Each item on the PSC receives zero, one or two points, with the scores on all 35 items summed for the total score. The recommended cut-off score of 28 or higher means that he/she has more problems than most other children of that age. A positive score on either version of the PSC suggests the need for further evaluation. Studies had also indicated strong internal consistency (0.91–0.93) of the scale when the inter-item analysis was carried out [42]. In Botswana, Lowenthal et al. [43] found that it had an internal consistency was 0.87 for the PSC and 0.86 for the PSC -Youth.

#### Outcome

3. *The Health of the Nation Outcome Scales for Children and Adolescents (HoNOSCA)* [44] **–** The HoNOSCA is a measure of outcome for use in child and adolescent mental health services focusing on general health and social functioning. The clinician measure is a 15-item questionnaire, to be completed by practitioners to indicate the severity of each problem, on a scale of 0–4. The measure made up of two sections: the first section consists of 13 items relating to different types of problems; the second consists of two items relating the parent or young person’s knowledge of the nature of the young person’s difficulties and their information about the services available. The self-report and caregiver versions consist of 13 items each, which correspond to the clinician measure – items 1–13. The total score of the HoNOSCA ranges from 0 to 52. It is a sum of the first 13 scales, representing the overall severity of the mental health problems experienced by the child or adolescent. The HoNOSCA is not considered to be a unidimensional scale with the initial study finding low intercorrelations between items [44]. The intraclass correlation coefficient (ICC) for the HoNOSCA total score was 0.81 (single scales 0.47–0.96) [45]. The HoNOSCA will be completed by all groups (the adolescent, caregiver, and clinician) at the intake stage. During the follow-up sessions, only the adolescents and clinician will complete the forms.
4. *Children’s Global Assessment Scale (CGAS)* [46] ***–*** This is a measure of assessing the severity of psychiatric disturbance and social disability. It will be used to provide a brief, stand-alone assessment of the patient’s global functioning before and after initiating treatment. The child or young person is given a single score between 1 (lowest functioning) to 100 (excellent functioning), based on a clinician’s assessment of a range of aspects related to a child’s psychological and social functioning. Reliability studies reported adequate inter-rater reliability (r = 0.84) [47] and intraclass reliability (r = 0.73) [46].
5. *The Youth Outcome Questionnaire 2.1 (Y-OQ-2.01)* [48] – is used as a measure of psychosocial distress to track treatment outcomes. The Y-OQ-2.01 is a parent-report measure of treatment progress for children and adolescents, aged 4–17 years old, receiving mental health services. It follows the measure of outcome explicitly and tracks change during treatment. It was constructed to be brief, sensitive to change over short periods, and maintain validity and reliability at a high level. The Y-OQ-2.01 consists of 64 items, rated on a 5-point Likert scale, which has available options from 0 (never), 1 (rarely), 2 (sometimes), 3 (frequently), 4 (almost always). The items comprise six subscales, which were found to be optimal in encapsulating change. The six domains included are interpersonal distress, somatic, interpersonal relations, social problems, behavioral dysfunction, and critical items. The Y-OQ-2.01 makes use of a cut-off score. To identify whether a child or adolescent is within the clinical or normal range, a score of 46 distinguishes the cut-off. All ratings above the cut-off are in the clinical range, while all scores below the cut-off are in the sub-clinical or normal range [49]. Internal consistency estimates of the total score across different studies were strong at .94 and .95, respectively [50, 51].
6. *The Youth Outcome Questionnaire Self-report version (Y-OQ-SR)* [52] – The YOQ-SR is a parallel version (60 items) of the Y-OQ intended for adolescents aged 12–18 years old. The Y-OQ-SR is also appropriate to administer at intake and before each weekly therapy session. The cut-off score for the Y-OQ SR-2.0 is 47 [52]. The Y-OQ-SR has demonstrated reliability, including strong internal consistency (.95) in previous evaluations [52, 53].

#### Therapeutic alliance

7. *The Therapeutic Alliance Scale for Children-revised (TASC-r)* [54] – This is a 12-item Likert scale administered to the therapist and adolescent (12 years and older) and used as a measure of therapeutic alliance across the treatment. It measures agreement between the therapist and the youth regarding the goals of therapy (6 items) and youth affect towards the therapist (6 items). The two versions of the TASC are parallel versions of each other. The questions are the same on each form but adjusted for the appropriate person completing the form (e.g., “I liked spending time with my therapist,” “The child likes spending time with you, the therapist”). Each item is rated on a 4-point scale ranging from 1 (not at all) to 4 (very much), with a total score calculated based on a composite of all 12 items and can range from 12 to 48, with higher scores indicating stronger therapeutic alliance. The TASC has demonstrated adequate internal consistency reliability (a = .72 to .74) in previous investigations [54, 55].
8. *Semi-structured interview guide*. We will carry out face-to-face interviews with all the groups (adolescents, caregivers, and clinicians). The interview participants will be selected randomly from those who already recruited into the study. Some of the questions for adolescents include: ‘*How would you describe the relationship between you and the therapist?’, ‘What type of support did you receive from them?’, ‘Did you feel heard, accepted, and in what ways?’, ‘How would you describe the process of counseling for your current problems….did you find it useful?’*. Caregivers: ‘*How would you describe the relationship between you and your child’s therapist?', 'Did they actively include you?’,‘Describe the type of support you and your family gave your child during this process?’, ‘Would you consider this process beneficial for the adolescent?’.* For the therapist: *‘How would you describe the relationship between you and the adolescent (and their caregiver)?’, ‘How would you describe the experience of building the therapeutic relationship with the adolescent/s?’, ‘Are their challenges and how do you mitigate them for the benefit of the adolescent?’, ‘What is the role of the caregiver, and how do you use them for the benefit of the process?’.*

### Data collection procedures

The study collection period will last approximately 12 months. During this time, all new patients at the clinic will be invited to participate, and all parents consenting for their children to participate in the study will fill out the researcher-designed demographic questionnaire, the PSC, and HoNOSCA. The children will fill out the demographic questionnaire, PSC-Y and Y-OQ-SR, while the therapists will fill out the demographic questionnaire, the HoNOSCA and CGAS. The child and the therapist will fill out the respective versions of the TASC-r at the end of the session. Each follow-up session will begin with caregivers and children completing the Y-OC-2.01 and Y-OC-SR, which will be used as baselines against which to evaluate the outcomes of the subsequent sessions, as well as a tracking tool to find the groups that are of challenge in therapy. The children and therapists will also fill out the TASC-r to give insight into the therapeutic alliance. These assessments take approximately 30–45 min for the participants to fill (see Table 1 for a detailed schedule of assessments).

**Table 1:** Assessment schedule

	***Parent***	***Adolescent***	***Clinician***
***First session***	• Demographic questionnaire • PSC, • HoNOSCA	• PSC-Y • HoNOSCA • Y-OQ-SR (12-17 yrs)	• HoNOSCA • CGAS
***Follow-up – before each session***	• Y-OC-2.01 • HoNOSCA	• Y-OQ-SR (12-17 yrs) • HoNOSCA	
***Follow-up - after each session***		• TASC-r	• CGAS • TASC-r • HoNOSCA

LMICs have and still benefit from frameworks already laid down for CAMH research. However, investigation of any kind in the field of mental health must be cognizant of cultural nuances [56, 57], more so when it comes to understanding and managing these disorders. One of the challenges experienced in the LMIC contexts, like Kenya, is the use of western designed diagnostic tools and interventions which are subject to influences from local settings and cultural differences, which may affect effectiveness in unpredictable ways. Despite evidence showing the efficacy and effectiveness of these interventions in the West, contextualized evidence is lacking. The study tools will be pre-tested in a similar environment to assess their suitability for use in our context. We will carry out a pre-test whose aim is to evaluate the chosen measures to see if they are easily understood by the different groups of participants, the time it takes to complete the questionnaires as well as the practicability of collecting session-wise data. Reliability and validity analysis will be conducted, and measures that are found not suitable will be removed before starting the data collection.

We will carry out qualitative interviews with a sample of the participants (children, caregivers, and clinicians). The discussions will focus on understanding what processes treatment operates and which facets may be particularly influential to treatment outcomes in this group. There are three types of stakeholders whose perspectives are essential to this work—the adolescent participants, their caregivers, and mental health providers at different cadres (nurses, intern psychologists at bachelors, masters, and Ph.D. levels; psychiatrists at consultant or master’s level). As stakeholders in the treatment process, it is essential to understand each experience of mental health problems as research has shown that how an individual conceptualizes a mental disorder influences the treatment and therapeutic change [58, 59]. The interviews aim to explore the perceptions and mechanisms which influence change to take place beyond the context of psychotherapy. Adolescents and caregiver voices in seeking these services will be of relevance in understanding issues around therapeutic alliance, mental health, and quality of services.

### Training procedures

We will recruit research assistants to assist in the collection of quantitative data. The research assistants will be individuals who have attained a minimum level of tertiary education (either certificate, diploma, or bachelors’ degree). They will participate in a two-day training whose purpose will be to create awareness and understanding of the formal ethical considerations and to ensure each participant gives appropriate written consent. The training will also familiarize them with the data collection process focusing on the tools to be used, what they assess, and their use, as many of the measures will be repeated in each session. If at any point during the data collection period we find that there are discrepancies within the data (such as missing data), we will retrain them to ensure quality data collection and quality data.

### Quality assurance procedures

The researcher will monitor the quality of data (e.g., are responses legible, no missing data) throughout the study. If the researcher identifies a lot of missing data that may compromise the ability to analyze the data later, additional training will be given to the research assistants to ensure the quality of data collected. We will also keep a log of any unexpected or adverse events encountered throughout the period.

### Ethical considerations

This study has undergone an ethical review from the Kenyatta National Hospital/University of Nairobi/ Ethics Research Committee (KNH/UoN ERC P761/11/2018), and clearance from the mental health department to research the adolescent clinic will also be sought.

Once we have all the approvals, we will approach the participants at the adolescent clinic. We will explain the purpose and objectives of the study to those contacted and allow them to seek clarification. We will inform them that their participation is voluntary, and the information collected is for the study alone. Those who refuse to participate or withdraw at any stage will not be penalized, and their withdrawal will not influence the services they seek at the institution.

Participants who meet the inclusion criteria and are willing to participate will be included in the study. The study will not discriminate against any political affiliations, gender, race, sexual orientation, or physical impairment. As mentioned earlier, only adolescents with a history of severe physical or mental impairments (blindness, deafness, autism) will be excluded from the study.

We will assure the participants that their data will be kept confidential and will only be used for research purposes, as well as to improve treatment services for future patients. The study will maintain the anonymity of the participants. There will be no personal identifiers on the questionnaires, only serial codes, and this will ensure that no participant can be traced. Completed surveys will be kept in a secure password-protected safe.

The major participants in this study are adolescents. We will train the research team on ethical issues around engagement and data collection with children and young people. Priority will be on issues of informed consent, voluntary participation, discrimination, the confidentiality of information collected from interviews to protect their anonymity. Any adolescent or caregiver who expresses distress will be routed to the psychiatric nurse or psychiatrist on duty in the department of mental health for immediate attention. We will share with the Department Chair any instances of child abuse or violation of rights, and later provide to the children’s officer.

### Data analysis

The quantitative measures have not previously been validated in the Kenyan context. Of the measures chosen, only the PSC has been validated in the sub-Saharan context; in Botswana [43]. We aim to validate the measures during the pre-test phase to assess their suitability for use in this population. To investigate the reproducibility and consistency of the tools, reliability coefficients, as measured by Cronbach’s alpha, will be calculated. We will calculate concurrent and convergent validity using Spearman’s correlation analysis. The relationship between scores of outcomes (HoNOSCA and Y-OQ-SR) and therapeutic alliance (TASC-r) will be investigated to determine the magnitude of the relationship between the measures. Convergent and concurrent validity requires that similar scores should correlate.

We expect our sample will be of mixed diagnosis, treated with a mix of therapeutic interventions, mostly of cognitive-behavioral or psychodynamic orientations. Data analyses will be conducted in SPSS version 26 using a stepped approach. First, we will test the process and outcome data for normality, skew kurtosis, and outliers. Second, we will conduct a descriptive analysis of the data using frequencies and percentages. We will then carry out bivariate analyses to examine the association between process variables and outcome variables. The process variables identified to be associated with the outcome will be entered into a regression to determine to what extent these factors predict the outcome when pre-treatment scores are controlled. We will also carry out within patient and between patient analysis to identify process factors that may affect the outcome in this cohort.

Longitudinal symptom change trajectories will be analyzed using latent trajectory/growth mixture modeling [60]. We expect that this analysis will result in at least one class of patients who are not improving. We will also test explanatory variables predicting latent class membership to learn which patients are not getting optimal results from treatment. Some of the explanatory variables of interest include patient psychiatric diagnosis, proposed therapy, number of sessions, the time between sessions, level of clinician’s education will be tested. Additionally, the outcome for these groups will be compared to published outcome data from international research, using a benchmarking approach [61, 62] to see if they would be expected to improve more.

We will carry out a network analysis to assess the representation of relationships and patterns of interaction among outcomes within the network. Network models are used as a sparse representation of the joint distribution of observed indicators. As such, these models show great promise in psychometrics by providing a perspective that complements latent variable modeling. Network modeling highlights the variance that is unique to pairs of variables [63]. This analysis will provide tools to understand outcome occurrence patterns in adolescents dealing with various problems and are receiving care. This analysis will highlight the possible interactions between outcome and therapeutic alliance as assessed by both the adolescent and clinician, indicating the presence of covariances that cannot be explained by any other variable in the model and can represent—possibly reciprocal—causal relationships.

To understand how adolescents, caregivers, and clinicians conceptualize mental health problems and attribute their difficulties will be studied using qualitative interviews. Interviews will also be conducted with clinicians to investigate ways they have found to adapt psychotherapy techniques to patients’ cultural expectations and beliefs. We will transcribe the recorded material verbatim, and where necessary translate them. We will use interpretative phenomenological analysis (IPA) [64] to analyze our data. IPA focuses on magnifying the subjective experiences of participants. It also focuses on a detailed, in-depth exploration of individual lessons, allowing us to make sense of the participants’ experiences of therapy and the therapeutic process [65]. We will use IPA to tease out thematic categories relevant to the experience and context of adolescents receiving psychotherapy in a Kenyan institution. This information will be on how the various participant groups interpret the therapeutic process, the therapeutic relationship, as well as how they influence the adolescent’s well-being while participating in the therapeutic process.

### Expected outcomes and benefits of the study

Child and adolescent mental health evaluation research is an underdeveloped field in Kenya. There is a lack of supporting policy, specialized services, and human resource challenges that perpetuate this continued neglect. Other than the treatment gap, one of the most critical issues in mental health services research is the difference between what we know about effective treatment, what is provided to and experienced by consumers in routine care in community practice settings. This research is essential as it will help us highlight the relative weaknesses and strengths of child psychotherapy conducted in Kenya, allowing us to use this information to improve mental health care and treatment in Kenya and hopefully other African countries.

Due to the paucity of information in this area, findings from this study will fill gaps in the literature regarding the effectiveness of therapy in routine clinical care for the management of child and adolescent mental disorders and the various factors that influence outcomes. An obvious goal of treatment is to optimize therapeutic change; therefore, by understanding the processes that account for therapeutic change in this cultural context, we will be better able to foster and maximize treatment outcomes for patients. Finally, this research will improve our understanding of how therapy works in our context by helping us identify moderators or mediators of treatment on which the effectiveness of a given treatment may depend.

## Data Availability

Not applicable.

## Abbreviations

CAMH: Child and adolescent mental health
DSM-V: Diagnostic and Statistical Manual of Mental Disorders
PSC: Paediatrics Symptom Checklist (parent version)
PSC-Y: Paediatrics Symptom Checklist - Youth version
HoNOSCA: Health of the Nation Outcome Scales for Children and Adolescents
CGAS: Children’s Global Assessment Scale
Y-OQ-SR: Youth Outcome Questionnaire Self-report version
Y-OQ-2.01: Youth Outcome Questionnaire 2.01
TASC-r: Therapeutic Alliance Scale for Children-revised
